# Scaffolding patient agency: Conceptualising readers’ cognitive work in the comic gutter

**DOI:** 10.1177/13634593241290184

**Published:** 2024-10-17

**Authors:** Amanda Roberts

**Affiliations:** University of Hertfordshire School of Law and Education, UK

**Keywords:** comics, gutter, imagination, life-limiting illness, liminality, reflexivity

## Abstract

A life-limiting illness can erode an individual’s positive sense of self. Storytelling can help counteract this, through scaffolding patients’ agency and supporting them in acting to change something which matters to them. This article explains how visual stories – comics – are used within the PATCHATT intervention to support the redevelopment of a person’s agential self. Through the provision of a conceptual map, this article explores the gutter as a liminal space, arguing for the importance of the deep reader engagement which takes place there. It uses Bob’s comic, a story used within PATCHATT, to explore how reflexivity and imagination work together within the liminal space of the gutter to stimulate and enhance palliative care patients’ agential change leadership. It concludes by considering the implications of the argument put forward for palliative care practice.

## Introduction

The complex impact of a life-limiting illness is well-documented (Lawton, 2000; Martin, 2016; Palmer-Wackerly et al., 2018), with old ways of being and seeing challenged by a diminishing sense of positive identity. A particularly distressing effect for individuals is declining agency, a diminishing capacity to positively impact on their personal and wider world (Exley and Letherby, 2001). Storytelling has been found to support individuals in re-claiming their agency (Bruner, 2002). Stories can be written in words or told in other verbal or visual forms (Bate et al., 2004). This article explores how visual stories – comics – are used within the PATCHATT intervention to support patients with a terminal illness. Whilst definitions of comics are much debated (see, e.g. Cook, 2011; Meskin, 2007), I follow McCloud (1993: 9) in seeing comics as ‘juxtaposed pictorial and other images in deliberate sequence’. The role of the gutter, the space between comic panels, in supporting the reader’s role in creating comics’ meaning is well-established. In this article I extend this understanding through offering a conceptual map of the elements involved in this meaning-making and how they interact. I propose that it the meaning-making facilitated by this interaction that enables patients with a life-limiting illness to explore and shape their own narrative and, in so doing, re-discover their empowered and autonomous self. I begin with a discussion of how this self is impacted by terminal illness.

## The impact of terminal illness on identity

Saunders et al.’s (1995: 45) concept of ‘total pain’ perfectly encapsulates the physical, mental, social and spiritual suffering experienced by many at the end of life. The term ‘total’ here evokes multi-faceted, all-encompassing suffering. A positive sense of self can be severely and incessantly challenged by such an onslaught (Broom and Cavenagh, 2011). Bury (1982) sees this challenge as emerging from three forms of disruption: of a person’s assumptions, their biography and their resource-base. Here, their view of who they are, who they will move on to be and who will support them in so being, all come under attack.

The idea of disruption relies on a popular view of identity as fixed and inflexible – we know our own and others’ characteristics and do not expect either to change (Jenkins, 1996). Living with a life-limiting illness challenges this position (Carlander et al., 2011), supporting Erikson’s (1975) alternative conceptualisation of identity as a work in progress. Here, the ravages to self which illness brings make the passive term ‘identity’ seem redundant. Instead, repeated instances of ‘identification’ (Brubaker and Cooper, 2000) are needed to understand our current and potential selves. Even when terminally ill, considering who we now are allows us to reject externally imposed expectations and boundaries and continue to realise our potential through productive lives (Charmaz, 2006). Maslow (1943) terms this self-actualisation, a state placed high in his hierarchy of needs.

Whilst the dominant medical approach to alleviating total pain focuses on Maslow’s (1943) lower levels of physiological and safety concerns, an alternative argument suggests the importance of intervention at higher levels which can support the development of agency (Jackson et al., 2014; Zalenski and Raspa, 2006). I use the term agency here to describe the aspect of the human condition which enables us to make a difference to the world around us. Some may refer to this as intentionality, that is, ‘the will or intention to make a difference, with and through others, which leads to action’ (Woods and Roberts, 2018: 7). For Frankl (2004: 105), such agency is driven by ‘man’s search for meaning’, the unique and specific value base which each individual acts to satisfy. An individual’s identity and agency are thus intertwined, supporting their purposeful changemaking in the social world (Roberts, 2022). Frost (2020) suggests that we need to focus not only on strengthening agency but also on working with it, providing scaffolding to enable it to flourish. Bruner (2002) offers storytelling as one such scaffold, taken up in the development of the PATCHATT intervention.

## The PATCHATT intervention

The PATCHATT (Patients Changing Things Together) (Roberts, 2022) intervention was developed by a group comprising patients, carers, volunteers, clinicians and academics (https://www.patchatt.co.uk/). A response to COVID lockdowns, patient members of a hospice day therapy group initially came together online to provide peer support. Post-Covid, the importance of helping one another to identify issues in life and take steps to address them was surfaced by group members. They decided to try to capture in a series of steps the support for action which the group had provided. A development group was formed, clinicians and others were invited to join and the PATCHATT intervention was born.

The intervention aims to enable patients with a life-limiting illness to support one another to make a difference to something which matters to them. Participants join a weekly support group, for 6 weeks. Volunteer facilitators guide participants through a series of activities which help them to articulate what matters to them, plan actions they will take to make the difference they wish to see and review weekly the progress they make towards achieving their goals. In the proof-of-concept run, participants struggled to see how they could move from the identification of what mattered to them to the development of a plan for change. Storytelling’s potential to support change leadership is well-established (VanDeCarr, 2015; Wittmayer et al., 2015), as is its particular benefit to those living with a life-limiting illness (Frank, 2013). The PATCHATT development group therefore developed exemplar stories of change leadership by adults with chronic illness, to stimulate discussion and reflection.

## Scaffolding agency through storytelling

A story is an organised account of events and experiences, constructed to share a specific meaning with a reader or listener (Anglin et al., 2023). The cathartic value of storytelling has been long acknowledged (Lieblich, 2013), with its’ potential to boost the development of a positive identity particularly relevant for those with a life-limiting illness (Bruner, 1996; Clandinin et al., 2009; Ricoeur, 1980). I would argue that this comes from storytelling’s power to support agential action.

McAdams (1996) early understanding of storytelling’s influence on agency has been extended by subsequent studies (see, e.g. Chen, 2012; Sakalys, 2003). Some story types have a particular capacity to support movement towards this sense of positive self. Quest narratives, for example, where illness acts as a call to action (ElShafie, 2018), propel the hero into a new context for personally meaningful activity (Frank, 2013), developing agency and self-efficacy (De Meyer et al., 2021; Southall, 2011). Bruner (2002: 65) summarises this succinctly – ‘self-making is a narrative art’. Stories’ call to action can stimulate change at a variety of levels. Persuasive stories, for example, can lead us to change our way of thinking, inspire us to overcome challenges (Goodchild et al., 2017) and to articulate individual goals (Young and Rodriguez, 2006). Narrative’s potential for influencing wider community action (Mourik et al., 2021) is exemplified through counter stories which, through contesting accepted norms and practices (Abma, 2003), support patients’ change-making.

Enabling patients to imagine an alternative future is key to scaffolding their potential to make the changes they desire (Essebo, 2022; Mourik et al., 2021; Schedlitzki et al., 2015). Patient evaluation of the original PATCHATT stories’ potential to support this imaginative leap was discouraging however. Echoing Frosh (2002, cited in Leitch, 2006), patients found the written text overcomplicated and inadequate to convey complex emotions and beliefs. Encouraged by Woods (2011) belief in the accessibility of non-linguistic forms of medical narrative such as music and art, we therefore began to produce the PATCHATT stories in comic form, relying on the positive impact of a combination of visuals and texts (Pedri, 2023).

## Comics’ affordances in the field of health

Graphic medicine stories – also called narratives and pathographies - use comics to communicate the experiences of multiple actors – patients, carers, families and healthcare providers (Williams, 2012; Wombles, 2021) – including its darker side (Glazer, 2015). Medical education and practice benefit from comics’ ability to highlight and develop key skills of communication, empathy and creativity (Adamidis et al., 2022) and how they help people visualise alternative perspectives through portraying both experience and emotion (Sinervo and Freedman, 2022). Comics thus support healthcare professionals in developing the ability to respond to the stories of individuals, to practice the empathic medicine patients deserve (Charon, 2021). Through understanding personal narratives, dominant medical discourses can be challenged (Krishnan and Jha, 2022) and the voices of the marginalised can be heard (Czerwiec et al., 2020).

It is the use of comics by patients which is the focus of this article. Comics’ use of spatial representation of time, visual metaphors and facial and body gestures enable patients to explore their unique yet shared (Green and Myers, 2010; Krishnan and Jha, 2022) and embodied (Venkatesan and Ancy, 2023) illness experience. Such exploration supports subsequent patient decision-making (McNicol, 2017) whilst promoting selfhood and dignity (Czerwiec and Huang, 2017). Differing comic representations of illness and the total pain which accompanies it (Czerwiec et al., 2020) provide a way to counter suggestions of patient culpability in illness (Sontag, 1978). Moreover, they offer patients a way to re-assert their ability to effect change (Venkatesan and Saji, 2021). The particular way in which comics scaffold such agency merits further exploration.

## Scaffolding agency through comics

Comics have long been used to scaffold agency. The quest narratives of traditional superhero comics, for example, exemplify resistance in the face of challenge. More recently, the advent of digital comics has encouraged readers’ social awareness (Shelton, 2014), challenging mainstream culture and allowing the articulation of otherwise oppressed voices (Nayek, 2021). In community-member produced grassroots comics, individuals are given free-rein to persuade others of their viewpoint (Gavsie, 2017), potentially stimulating community-led change (Anand and Anand, 2019; Pakalen and Sharma, 2007).

Although there are common characteristics in comics supporting change (Anglin et al., 2023; Beck, 2018; ElShafie, 2018) the way in which the reader reads the texts appears more powerful than particular story elements (Alawafi et al., 2021; Ricoeur, 1984). Some appreciation of comic reading approaches is therefore useful.

In comics, a story is made up of a complex arrangement of thoughts, actions and ideas, conveyed in a sequenced arrangement, often separated by panels. Time, a key structuring agent in comics, is communicated, and often manipulated, through panels which explore a particular action, emotion or thought process (Eisner, 1985). The repetition of icons and recognisible symbols such as words and images work together to form the comics’ storytelling language (Eisner, 1985). Particular story elements can be conveyed both through large gestures – such as the colouring of a whole panel black – and small details, such the raising of a character’s eyebrow (Eisner, 1985).

This story structure supports alternative reading strategies to those used for text alone, both for the panels themselves and the space between them. In comics, textual and visual languages combine (Cohn, 2023), expanding the reader’s opportunity to engage with the story through controlling the pace of their eye movements across the page of panels (Dicks, 2015). The convention of right to left engagement sequence can be broken, with the reader filling in details from their own experience (Eisner, 1985).

Green and Myers (2010) explanation of the impact of alternate brain information processing systems activated by text and images further illuminates comics’ potential to initiate new thinking, with the melding of text and image allowing connections between new information and existing knowledge. McCloud’s (1993) suggestion that this prompts readers to engage with comics in an agential way is extended by Ricoeur (1984). He argues that storytelling’s powers of persuasion arise not from an individual’s solo cognitive connections with expected elements but from a reciprocal social process of action and reaction. This unique link between teller and listener through conjoint activity in the sense-making process provides the power for change (Shapiro, 1993, in Sakalys, 2003). McCloud (1993) suggests the space required for such reader agency is provided by the potential pause in space and time between panels, the gutter, which offers the reader the opportunity to understand the story and make meaning from it for their own unique situation.

## The invitational power of the gutter

Blank spaces are a feature of various storytelling forms. Novels, for example, are often divided into chapters, with blank space left at the end of one page before a new chapter begins. Whilst such spaces can be used for reflection or to signify a lapse in time which a reader must fill, it is more usual to imagine them as convenient stopping points on the journey through the whole text. Blank spaces around visual stories are also common. A hung painting, for example, has a space around the canvas or frame, enclosing the picture, positioning it as distinct from the next painting. Within comics, however, where text and pictures combine, storytelling panels often represent unconnected moments in space and time (McCloud, 1993).

McCloud (1993: 88) argues that the gutter offers the reader the opportunity to make the transition between one space and time and another, to close temporal and spatial gaps in order to make sense of the panel’s story, hence his use of the term ‘closure’. In considering what must happen in time and space to allow subsequent panels to make sense, the reader reflects not only on the story being presented (Green and Myers, 2010) but the subtext, what the story means to them (Duncan, 1999). This joint co-creation process, it is argued, is needed to enable the story to retain full value or even, in some cases, to make sense (Eisner, 1985; Wolk, 2007). The logic of this argument is contested by Hatfield (2022), highlighting that whilst McCloud (1993) argues for the empowerment of readers, he also foregrounds the meaning-shaping properties of the authorial use of image and text. However, the power of this argument relies on a reader consciously reading and making meaning. Cohn (2023) challenges this view, developing Selden et al.’s (2005) debate to explore how the unconscious process of the mind involved in reading comics makes sense of incomplete stories. Supported by Davies (2022), this argument is strengthened when considering how readers engage with abstract comics, where notions of beginning and end lose relevance. Cohn (2023) suggests readers here respond to chunks of meaning. Gavaler and Beavers (2020) understanding of closure as a subset of inference would support this theory.

## Meaning-making in the gutter – A conceptual map

Whilst the argument for meaning-creation in the gutter is strongly supported then, the process by which this meaning is made is less well understood. This article proposes the interaction of liminality, reflexivity and imagination to be key to this meaning-making. A conceptual map is offered to illustrate this interaction, with an example of a PATCHATT comic used to explore the map’s explanatory value in practice.

### Defining the map’s elements – Liminality, reflexivity and imagination

With its roots in anthropology, liminality refers to the ambiguity or uncertainness which occurs in the middle stages of rites of passage or change processes which may involve the individual in a reconstruction of identity (McKechnie et al., 2010). The concept of liminality has been used to explore palliative care doctors’ and patients’ understanding and experience of illness and healthcare (see e.g. McKechnie et al., 2010; O’Callaghan et al., 2020).

Reflexivity has its roots in social theory. It is a fundamental human quality which allows us to reflect on how we think and what affects it. This process of self-awareness enables us to critique our natural interpretation of life through reference to previous experience (Siraj-Blatchford and Siraj-Blatchford, 1997). We can consider the assumptions we bring to our understanding of life and the stances we adopt (Holland et al., 1998), enhancing our understanding of ourselves, of others with whom we interact and of the social structures within which such interaction takes place. We then have the potential to change the way we think and act, to have agency over our social world (Verdonk, 2015).

Reflexivity has a key role in the development of identity through activity. For Goffman (1959), the reflexive process draws on a deeply held view of who we want to be and of the actions which will best move us in the direction of this desired self. Archer (2003) takes this further, examining the potential impact of reflexivity for individuals, society and the relations between them. A particular type of reflexivity, the ‘internal conversation’, is suggested as a scaffold for individual action-planning. Here, an internal dialogue offers individuals increasing agency in their lives.

By ‘imagination’ I am referring to the human faculty, often unconscious, to draw together what we have seen and understood and generate new stories and possibilities, not necessarily expressed through language (Asma, 2017). Here, our imagination combines with our narrative powers to support the cognitive leaps necessary to develop our own interpretation of a story (Bruner, 2002), melding known facts with self-conceived explanations (Kearns and Kearns, 2020).

### ‘Reading’ the conceptual map

Following an approach suggested by Grant (2003), this conceptual map is designed to allow concepts to be added sequentially in a layered approach. This approach allows for the discussion of each element and a justification of its place in the map. It also allows us to consider the impact of one element on another and the complex nature of the whole (Grant, 2003).

**Figure 1.**
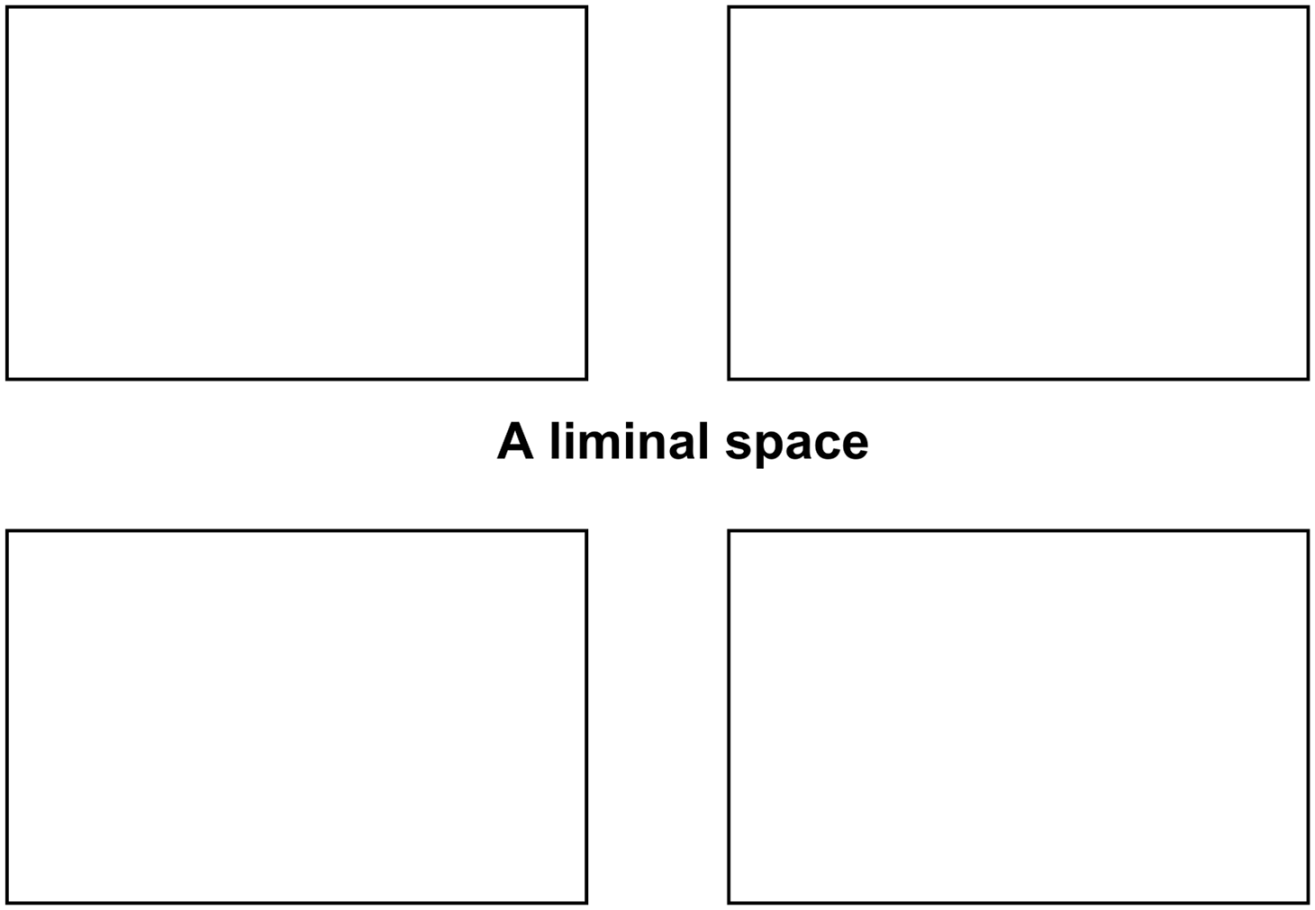
The first layer: a liminal space.

The first layer of the map introduces the concept of a liminal space (Figure 1).

**Figure 2.**
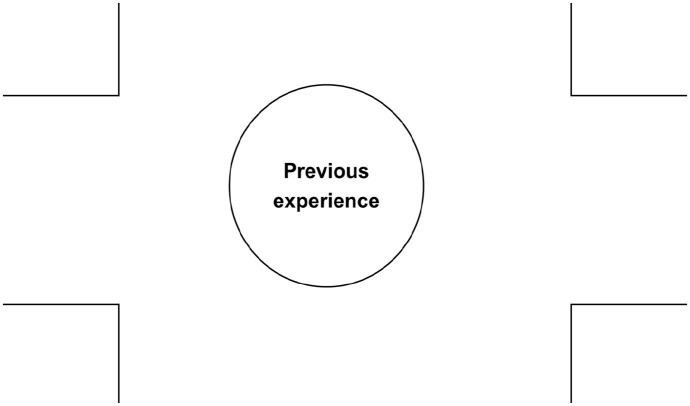
The second layer: introducing previous experience.

Here, the square boxes represent the comic panels, with the gutter represented by the white space between them. The gutter is conceived as a liminal space. Land et al. (2014) describe a liminal space as a place of transformation, where an individual’s previous frame of meaning can be re-considered and re-formulated. Conceptualising the gutter as a liminal space (Venkatesan and Saji, 2016) provides an opportunity for readers to rest and consider both the comic story and their own. It offers the potential for reviewing and revising viewpoints, a common precursor to change (Figure 2).

**Figure 3.**
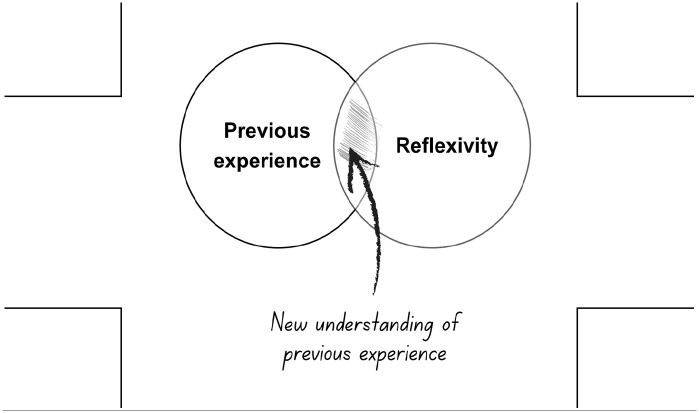
The third layer: introducing reflexivity.

The centre of the map has been enlarged in this second layer to show activity in the liminal space of the gutter. The reader brings their previous experience to this space, offering them an opportunity to see the connection between their own story and the comic story unfolding in the panels. It is this connection to the experience of others which enables new understandings of this previous experience to evolve (Figure 3).

**Figure 4.**
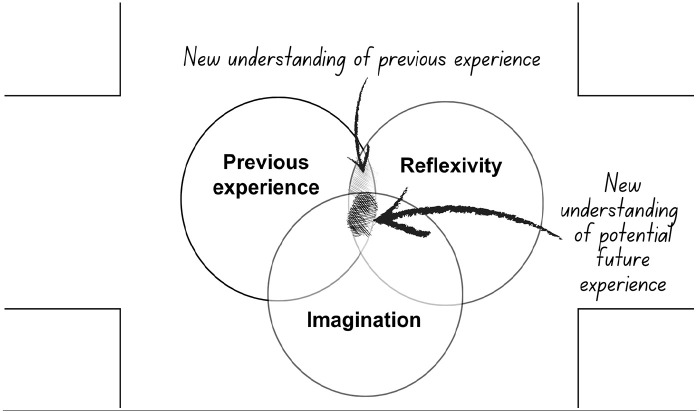
The fourth layer: introducing imagination.

This potential for renewed understanding is enhanced by reflexivity, introduced in the third layer of the map. The shaded intersection of the map illustrates where previous experience and reflexivity coincide. It is in this space that readers can develop self-awareness of their own thinking and what impacts on it. This enables them to understand their lived experience in a new light, stimulated by the unfolding story in the previous panel. Such new understanding paves the way for change (Figure 4).

The fourth layer of the map illustrates the impact which introducing imagination can bring. Wolk (2007) makes a persuasive argument that it is the reader’s imagination which is ignited by the blank spaces of the gutter, allowing them to make a coherent story (Iyyer et al., 2016). For adults with a life-limiting illness, this invitation to bring their own imagination into play is crucial. The imaginative process allows them to understand the relevance of the comics’ stories to their own situation, interpret this situation differently through a reflexive approach and use this new understanding as a catalyst for their own action. Their potential as agential actors in their own future broadens their sense of future possibilities.

## Working with the conceptual map: Bob’s comic

**Figure 5.**
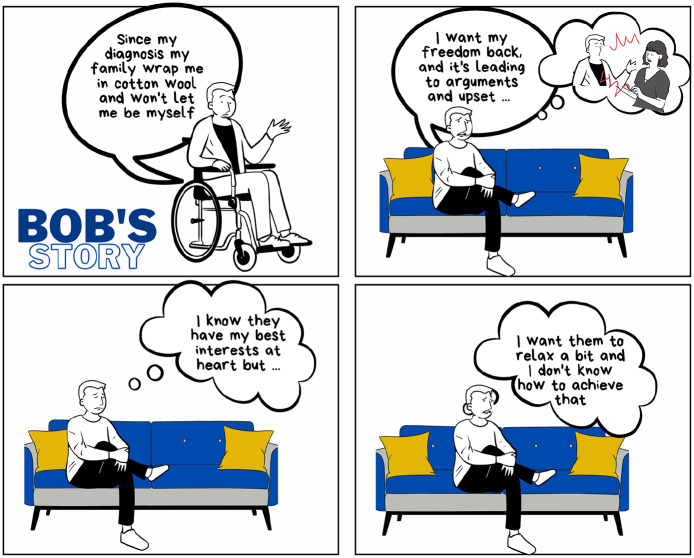
Bob’s story.

**Figure 6.**
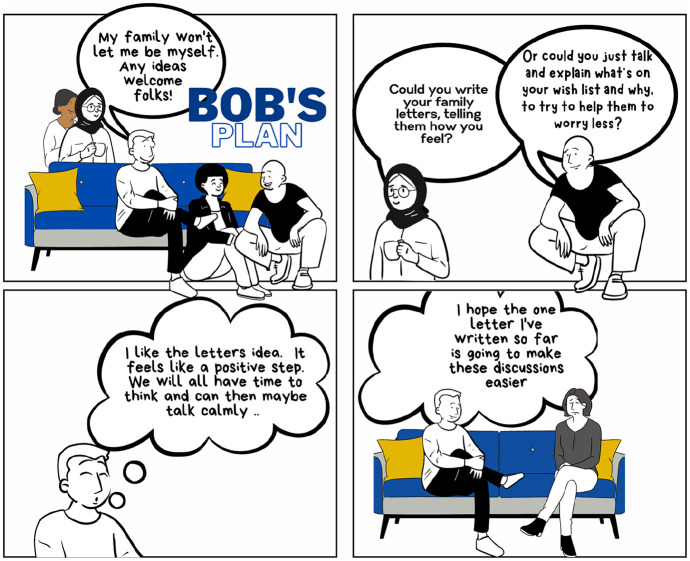
Bob’s plan.

**Figure 7.**
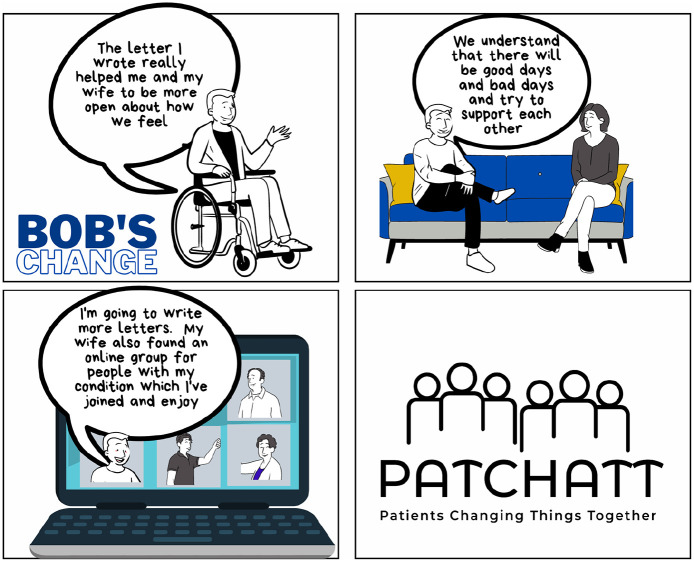
Bob’s change.

The comics made and used within the PATCHATT intervention tell the stories of Bob, Nita and Sylvie, all adults with a life-limiting illness, and how they came to make a difference to something which mattered to them. Comics were designed to exemplify the types of issues faced by patients with a life-limiting illness. We hoped that they might act as catalysts for personal reflection, discussion, planning and action, all crucial elements of the re-development of an agential self. Here I use Bob’s comic, shown in Figures 5–7, to interrogate the value of the conceptual map proposed above in explaining the emergence of this agential self in the liminal space of the gutter.

Bob’s comic is in three parts and is shared with group participants across three PATCHATT group sessions.

In the first comic strip, Bob shares his story, an issue common to palliative care patients – the difficulty of retaining a sense of independent self in the wake of a terminal diagnosis (Figure 5). Bob’s experience exemplifies all three of Bury’s (1982) disruptions. Bob’s comment in the first panel that his family ‘won’t let him be himself’ illustrates the depth of the challenge to identity brought about by chronic illness (Carlander et al., 2011). The remaining panels reveal Bob’s understanding of his family’s reactions, his desire to influence their reactions and his worry about not knowing how to achieve this.

Bob’s thinking takes place within the panels. However, the gutters between them require the reader to engage in cognitive work, to imaginatively empathise with Bob (Polack, 2017), not only to ‘close’ Bob’s story but also to develop a story of their own.

In this first strip, the gutter between panels 1 and 2 gives the reader time to pause, to slow down the story and think. They are asked to do the work of inferring that Bob’s changed health situation is causing him emotional pain. They can imagine the difference between his life before his diagnosis and his life now, and the impact of this change on his self-view. They can also bring their own previous experience into this space and examine it anew in the light of Bob’s reactions. Based on this active engagement and reflection, the reader moves onto panel 2, armed with the understanding necessary to interpret the additional detail of Bob’s situation and to apply it to their own. The nature of Bob’s disagreements with his wife are merely hinted at by the thought bubble in the second panel. It is for the reader to fill in the details, imagining both what might precipitate such confrontations and their deeper meaning and impact.

Between panels 2 and 3, Bob shifts from focussing on what he wants to trying to understand his family’s motivation in sheltering him. Accepting the authenticity of this cognitive leap requires the reader to imaginatively empathise with Bob (Polack, 2017), linking his story to their own in the intervening gutter. They are asked to understand the complex and contradictory feelings which Bob is expressing, and to empathise with him. This profound connection requires a reflexive response to the story shown in the panels. Reflexivity allows the reader to interrogate their own previous experience and more deeply understand the impact of a terminal diagnosis on loved ones. Bob’s visual and verbal confusion in panel 4 underlines this universality of experience (McCloud, 1993) and encourages the reader to stay with Bob on his journey and in so doing, with themself on theirs.

The second strip of Bob’s comic explores how he begins to work through these issues with his fellow PATCHATT group members and develop a plan of action (Figure 6).

Bob is helped to move forward with his issue by the support of his PATCHATT peers and the suggestions they make. Bob’s demeanour alters considerably in the second part of his comic. He seems enlivened by the ideas proposed and welcomes the opportunity for change. His subscription to a fixed view of identity evidenced in the first strip – ‘My family won’t let me be myself’ – is weakened here as his peers rely on Erikson’s (1975) alternative conceptualisation of identity as a work in progress to suggest how Bob can change his approach, be his current self and get what he now needs.

Positive reader activity is needed in the gutter between panels 3 and 4 if they are to replicate Bob’s success in reconsidering his own position and allow for the possible rebuilding of their own self through activity (Roberts, 2022). Reflexivity has already enabled them to consider past events. The introduction of imagination now allows them to consider if their own life has to be this way (Bruner, 2002). It is not enough here simply to acknowledge that Bob seems to have the potential to move forward. Instead, they must engage not only with the substance of the story as presented but also with its subtext, which readers must decode in an individual way and make their own (Duncan, 1999).

The imagination needed to support change leadership activity relies to some extent on reader empathy. The reader needs to see something in Bob’s story which they can connect with and make their own. The question of the root of this empathy is implicitly raised here. The concept of collective identities usefully addresses this. Jenkins (1996) argues that collective identities emphasise what people have in common and how this makes them similar to one another. Gee’s (2000) concept of affinity identity is similarly illuminative, emphasising how the practices we undertake mark us out as a member or not a member of a particular group. In PATCHATT, the readers of Bob’s comic have a collective identity as adults with a life-limiting illness. However, their responses to this illness will be multifaceted. Imagination can be brought into the liminal space provided by the gutter, where individuals can consider how they are like Bob and how they are different to him. The internal conversation comes into its own here (Archer, 2003), offering readers the opportunity to explore their taken-for-granted beliefs and behaviours and the way in which Bob’s story challenges or supports them.

In the final comic strip, Bob reflects on the action he took, the change he has managed to effect and on how this has impacted on himself and others (Figure 7).

Bob’s letter-writing appears to have had a positive impact on his life. This response to the call to action brought about by his illness (ElShafie, 2018) has led to more harmonious relationships with his wife and to his positive connection to others in a similar situation though an online support group. In taking control of his situation through acting to bring about change, he demonstrates how his intentionality, his will to make a difference (Woods and Roberts, 2018), can transform his situation. Bob appears to have moved forward not only in influencing how others see him but also in how he sees himself.

Bob’s change appears to be a consequence of his re-imagining of his situation and the confirmation of his ability to act to make a change. The liminal space between panels 1 and 2 invites the reader to delve into the ambiguity around both Bob’s capacity for change-making and their own. The remaining comic panels provide evidence of the efficacy of Bob’s approach. Stimulated by Bob’s success, the reader can reflexively consider how they have interpreted their future potential as an agential being. They can draw upon their imagination to take the cognitive leap towards a more positive future and to consider how they might act to bring this desired future about. The ‘known-facts’ of Bob’s story can here be combined with self-conceived explanations of Bob’s potential and their own.

## Conclusion

The graphic medicine movement makes strong claims for how comics might support patients’ challenge to hegemonic views of medicine, illness, death and dying. This study of the use of comics within the PATCHATT intervention has given further evidence of these comic affordances. PATCHATT proof-of-concept participants provided clear testimony of the impact of the comics on their own capacity for change leadership and positive self-view. However, this article seeks to go beyond providing additional evidence for already well-argued positions. Instead, it proposes that it is the particular combination of liminal space, reflexivity and imagination which is the primary catalyst for reader agency. A conceptual map is offered to illustrate the relationship between these elements.

Three propositions support the development of this map. Firstly, the gutter between panels is a liminal space in which readers can change perspectives, relinquish familiar frames of reference and consider alternative interpretations of their situation. The claim that comics offer the reader the opportunity to be a central creative figure in storymaking is supported by this conceptualisation of the gutter as a place of transition. Whilst the degree of reader autonomy may be questioned, a conceptualisation of the reader as active agent holds. Secondly, the liminal space of the gutter supports the reader’s reflexive internal conversation, questioning accepted mores around illness, dying and death and their place within them. It is this internal conversation which underpins patients’ self-view as an agential being and supports planning for change-making activity. Thirdly, this internal conversation provides a vehicle for the imagination, allowing patients to draw together what they have seen and understood in the comics to construct and accept an alternative, positive version of their own self (Land et al., 2014). These new versions of the self can be tried out, turning points can be reached and new meanings noticed, whilst reflexive self-questioning supports the potential for self-change (Beech, 2011).

These propositions and the model which captures them have clear implications for palliative care practice. The need to develop innovative ways of providing palliative care is well-established (Byock, 2000), alongside the imperative to provide such innovations at community level (National Palliative and End of Life Care Partnership, 2021). The extended understanding of how comics might scaffold patient agency offered in this article suggests a way to enable patients to become agential actors in this additional palliative care provision. Robust practice-based evaluation of the PATCHATT intervention will give the vital evidence now needed of the validity of this argument.
